# Application of noble cerium-based anti-corrosion sealing coating approach applied on electrical insulators installed in industrial regions

**DOI:** 10.1098/rsos.211786

**Published:** 2022-04-06

**Authors:** Simpy Sanyal, Taeyong Kim, Matheus Rabelo, Duy Phong Pham, Junsin Yi

**Affiliations:** ^1^ Department of Electrical and Computer Engineering, Sungkyunkwan University, Suwon 16419, Republic of Korea; ^2^ Interdisciplinary Program in Photovoltaic System Engineering, Sungkyunkwan University, Suwon 16419, Republic of Korea; ^3^ College of Information and Communication Engineering, Sungkyunkwan University, Suwon 16419, Republic of Korea

**Keywords:** reliability, interface health, cerium, green inhibitor, pH, high-voltage insulator

## Abstract

Pin corrosion is a critical issue that causes premature rupture of high-voltage insulators. The development of efficient, defect-free, thermal resistive, hard, economical and environment-friendly sealing coating system is required to replace the current polymer-based highly toxic coatings for insulators. This study investigates the suitability of noble cerium (Ce)-based sealing coating film for use as an anti-corrosion coating for insulator pins installed in low-pH and highly corrosive sites. The coating bath parameters are optimized for the formation of a high-performance Ce-based protective sealing coating. After immersion in a 60 000 ppm CeCl_3_.7H_2_O sealing coating bath for 60 min, a Ce-rich and dense protective coating (24 µm) is formed on the pin surface. The life expectancy of the coated pin is 2.5 times higher than that of the non-coated galvanized pins. Electrochemical experiments and microstructural analysis demonstrate that Ce-based protective layers are suitable for long-term protection of high-voltage insulator pins in low-pH and high-corrosion-rate sites. We believe that this work would pave the way of developing ecofriendly anti-corrosion coatings for electric insulators and power industries.

## 1. Introduction

Insulators are among the key devices in electric power transmission systems. High-voltage insulators provide mechanical support and electrical isolation to conductors from the ground structure. Globally, 72% of overhead transmission lines use porcelain-suspension insulators, which have a demonstrably higher resistance to surface degradation and do not deform unless an external force is applied. A lifespan of up to 30–50 years can be guaranteed [1]. Additionally, they are manufactured from natural materials using a simple blending and curing mechanism, may be stored in dumps containing other waste and may serve as recycled material for the production of ceramics and similar products [2]. Insulator assemblies and fittings mostly use galvanized metal parts owing to their successful history of corrosion resistance in low to medium polluted sites. In industrial and marine sites, a contaminated environment exacerbates the risk of corrosive attack for insulators [3,4]. The corrosion rate increases dramatically for contaminated, low-pH industrial and marine sites with frequent rainfall. Owing to the higher corrosion rate, more metal corrodes in a shorter span, resulting in lower mechanical strength in insulators. During transient conditions, insulators are simultaneously subjected to corrosion attack and voltage stress. These circumstances lead to the formation of hard radial iron oxide struts, which evolve into insulator pins. Ultimately, these struts penetrate the pinhole and employ bursting pressure, which causes radial cracks in porcelain. Additionally, the corrosion of either pin may reduce the cross-section or mechanical strength, thus leading to a sudden ejection of the pin [5–9]. Insulator damage may result in sudden failure of transmission lines, thus raising safety and financial issues for power industries. The losses reported by global power industries because of corrosion were approximately 2–5% of the gross national product of any country.

The global corrosion inhibition coating market was reported to be worth USD 13.5 billion in 2020 with a predicted compound annual growth rate of approximately 4.3% from 2021 to 2027. In addition to cathodic protection, other prevention measures include (i) structural improvement by implementing pure zinc sleeves, U-shaped zinc rings, organic zinc sleeves, and anti-corrosive covers over the pins; (ii) use of corrosion-resistant steel materials for pins, and (iii) coating the pin surface with petroleum-based grease (maintenance practice used in certain installation sites) [10–14]. However, the zinc sleeves and ring approach can lead to shell fracture owing to the volume expansion of corrosion by-products. The use of anti-corrosive covers can inhibit electrolytic corrosion to a certain limit; however, it is ineffective against galvanic corrosion. Implementation of corrosion-resistant alloys for pins is an expensive alternative where the adjoining metals should also be compatible. Coating the pin surface with petroleum-based grease may be justified in certain low-corrosive installation sites. The room temperature vulcanizing (RTV) silicone coating, a popular recent strategy used by the ceramic insulator industry, has major advantages such as corrosion inhibition, hydrophobicity and self-cleaning in outdoor environments. However, it is highly hazardous to the eyes, skin and inhalation system, and is carcinogenic in nature. The use of such non-biodegradable polymers creates significant disposal issues and adverse ecological effects [15]. In recent years, awareness of health, environmental and ecological risks associated with polymer-based materials has increased. This has led to increased efforts in finding a replacement for polymer-based coatings for insulators. Cerium (Ce) is an abundant and economical rare earth metal. Ce-based oxides are potential environment-friendly materials for technological applications such as corrosion prevention, super-hydrophobic coatings, thermal barrier coatings, optical devices, catalysis, glass abrasives and microelectronics [16].

The corrosion inhibition mechanism of Ce salts involves the development of insoluble Ce oxides and hydroxides at the cathodes to retard the oxygen reduction reaction. The application of green Ce-based coatings over Al, Zn and Zn alloys and galvanized steel to suppress metal corrosion has been discussed in many recent studies [17–21]. In all previously published papers, the conversion coating was created by either immediately immersing the metal specimen in a corrosive solution containing rare earth salts or by immersing the specimen in a conversion coating bath containing rare earth metal prior to immersion in corrosive medium. Such a procedure reported fractures in the deposited layer and necessitates a post-treatment after coating development [18,20,22]. The present article adapts a noble three-step process for developing sealing coating on pin specimens. The development approach of the sealing coating makes it efficient, defect-free, thermal resistive, hard, economical and environment-friendly sealing coating system without any post-treatment requirement. However, the synthesis approach adapted in the article is noble and such green coatings have not been implemented in insulator hardware yet.

The article describes implementation of sealing coating on insulator pin. However, it can be implemented on other metal parts prone to corrosion in power industries in future. For the adaption of any coating technology by the insulator industry, reliability evaluation is essential. The desirable lifespan of the sealing coating should be at least 30–50 years, similar to that of high-voltage outdoor insulators. Simultaneously, the coating material should resist temperature variations and harsh outdoor environments. This article discusses the reliability evaluation of Ce sealing coatings applied on insulator pins installed at low-pH (3–4) and highly corrosive (8–12 µm yr^−1^) industrial sites. In this study, the Ce-based noble sealing coatings were deposited on insulator pins using an economical dip-coating process. The sealing coating bath parameters were optimized for the formation of a high-performance Ce-based protective sealing coating. Surface morphology analysis was assessed using an optical microscope (OM) and scanning electron microscope (SEM). The composition of the sealing coating, temperature resistance, crack resistance, hardness, adhesive strength and binding energy were analysed using an energy-dispersive spectroscope (EDS), SEM, tape tests, cohesive zone finite-element approach and X-ray photoelectron spectroscope (XPS). The anti-corrosion properties of the sealing coating were evaluated using zinc reduction measurements of the galvanized coating.

## 2. Experimental set-up

### 2.1. Sample preparation

The Ce-based sealing coatings were deposited on new 10.7 cm insulator pin samples coated with 180 µm of Zn. Multiple samples and corrosive media were considered for each set of experiments to evaluate the repeatability and reproducibility of the observed results. The samples were mechanically abraded with SiC-500 and SiC-1000 mesh papers. Consequently, the specimens were desmutted with acetone, isopropyl alcohol and deionized (DI) water for 10 min each in an ultrasonic bath. Between each step of the pre-treatment process, the specimens were rinsed in a DI water bath. Further, the specimens were provided with alkaline cleaning by Turco-6849 (20 vol%) at 50°C and rinsed in DI water for 2 min. The conversion sealing coating is formed in three steps. The specimens were submerged in an acidic solution (250 ml) comprising H_2_O_2_ (2.5 ml) and HNO_3_ (5 ml) for 30 min to begin the formation of a metal oxide structure on the metal surface. The H_2_O_2_ acidic solution has a pH of 3.5. The specimens were immersed in DI water for 30 min at a temperature of 90°C. The temperature was enhanced to induce oxide thickening. After oxide thickening, the specimens were heated to 150°C using a Thermo Scientific heating oven then quenched in a Ce coating bath for 10–60 min at 25°C for developing Ce sealing film over oxide film. The sealing coating bath contained 60 000 ppm CeCl_3_.7H_2_O in 500 ml DI water. The sealing layer's role is to infuse and seal the oxide layer. The specimens were washed in a DI water bath between each phase of the pre-treatment and coating procedure. After the coating process, the specimens were stored at room temperature for 24 h for drying in ambient air. To evaluate the inhibition characteristics of Ce salts, one bare and 10 coated specimens were dipped in an industrial corrosive medium for 30 days. The industrial corrosive media Sol-I1, Sol-I2, Sol-I3 and Sol-I4 were prepared by H_2_SO_4_: 0.5 ml/NaCl: 6 gm/HNO_3_: 0.3 ml; H_2_SO_4_: 0.7 ml/NaCl: 10 gm/HNO_3_: 0.5 ml; H_2_SO_4_: 0.7 ml/NaCl: 15 gm/HNO_3_: 0.7 ml; H_2_SO_4_: 1 ml/NaCl: 15 gm/HNO_3_: 1 ml, respectively. The corrosive solutions Sol-I1, Sol-I2, Sol -I3 and Sol -I4 were prepared by maintaining a pH between 3 and 4, and the corrosion rate was calculated as 8–12 µm yr^−1^. All the immersion tests were performed under the conditions provided by the American Society for Testing and Materials (ASTM) G 31–72 and (ASTM) G1 standard specifications. Table 1 details the composition of the inhibition and industrial corrosive media at 25°C.

**Table 1.** Composition of inhibition and industrial corrosive media.

solutions	type	condition	pH	corrosion rate
Sol-A	inhibition	Ce/60 000 ppm (30 gm)/10 min	N/A	
Sol-B		Ce/60 000 ppm (30 gm)/30 min		
Sol-C		Ce/60 000 ppm (30 gm)/60 min		
Sol-D		Ce/60 000 ppm (30 gm)/90 min		
Sol-I1	industrial	H_2_SO_4_: 0.5 ml/NaCl: 6 gm /HNO_3_: 0.3 ml	4.8	5.58
Sol-I2		H_2_SO_4_: 0.7 ml/NaCl: 10 gm/HNO_3_: 0.5 ml	3.99	8.94
Sol-I3		H_2_SO_4_: 0.7 ml/NaCl: 15 gm/HNO_3_: 0.7 ml	3.32	11.81
Sol-I4		H_2_SO_4_: 1 ml/NaCl: 15 gm/HNO_3_: 1 ml	3.05	12

The X-cut tape test was performed on the Ce-coated specimens dipped for 60 min in a Ce sealing coating bath to evaluate the adhesion strength of the sealing coating. Two cuts were made in the sealing coating film with a fine-edged cutting device approximately 40 mm long and 0.029 mm depth that intersected near their middle, making an angle of 30° and 45°. A pressure-sensitive transparent tape (40 mm long, 12 mm wide) with an adhesive peel strength of 6.34 N cm^−1^ was placed at the intersection of the cuts. The surface of the tape was rubbed firmly by applying pressure until the colour was uniform in appearance. Within 90 s of application, the tape was removed by seizing the free end and pulling back at an angle of 180°. The X-cut area was inspected to remove the sealing coating from the substrate. The same experiments were performed on the 10 Ce-coated specimens to evaluate adhesion. The tape test was performed in accordance with D3359 standards.

### 2.2. Measurements and characterizations

The thickness of the specimens was measured using an Elemetron thickness gauge meter to fix the sealing coating bath parameters. Five sets of insulator pin specimens were individually treated in a 60 000 ppm Ce coating bath for 10, 30, 60 and 90 min. That is, a total of 20 insulator pin specimens were immersed in a sealing coating bath, with five sets each for 10, 30, 60 and 90 min immersion times to observe coating thickness at various immersion times. All the 20 specimens were measured after treatment. The thickness of the deposited sealing coatings was measured for all the specimens to make the process more scalable and reproducible. The corrosion behaviour of the Ce-coated pins were evaluated by measuring the thickness of the bare and Ce-coated specimens using an Elemetron thickness gauge meter after dipping them in an industrial corrosive medium for 30 days. An OM (Korean Scientific Inc. UCMOS03100KPA) and SEM equipped with an EDS (FESEM III/EDS, JSM7500F) were used to analyse the surface morphologies and chemical configuration of the sealing coating at various coating bath parameters. The hardness of the film was measured using a Vickers microhardness tester (Shimadzu/HMW-2). The load was 90 mN and the loading time was 10 s. The measurement of each sample was performed at three points and the average hardness value was considered. In addition, the cohesive zone model (CZM) approach was implemented in COMSOL to evaluate the adhesive strength of the interface. XPS was performed using a fission instrument to measure the atomic concentration of elements on the surface of the specimens. The C 1s peak placed at 285 eV was selected as the reference for the internal binding energy. The base pressure in the ultra high voltage (UHV) chamber and spectra were measured at 10^−9^ mbar and 15 kV.

## 3. Results and discussions

**Figure 1.**
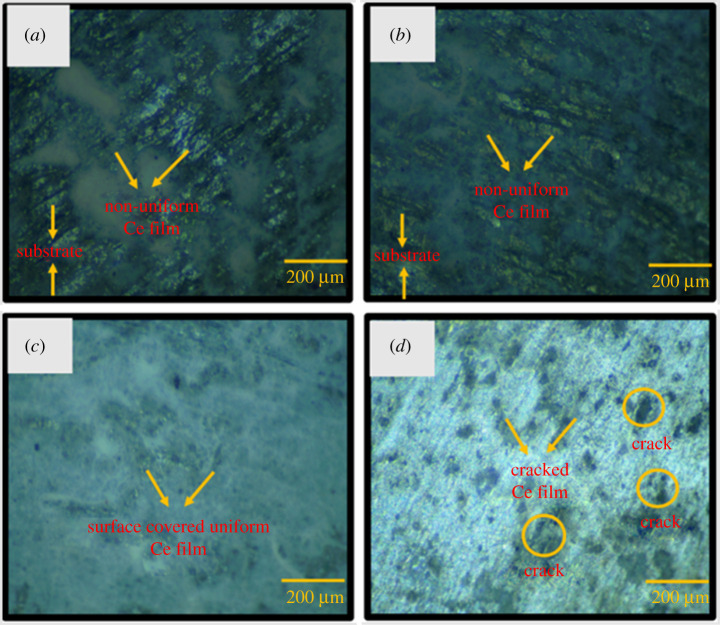
OM micrographs of Ce-coated specimens after different immersion periods: (*a*) specimen SA after 10 min, (*b*) specimen SB after 30 min, (*c*) specimen SC after 60 min and (*d*) specimen SD after 90 min.

Traits were assigned to the specimens after the samples were removed from the conversion bath and dried for 24 h. The Ce sealing coatings on the insulator pins attained after different immersion times in the Ce solution are presented in figure 1. Figure 1*a,b* shows the OM micrograph of Ce-coated galvanized steel pin specimens after 10 and 30 min of immersion in a 60 000 ppm CeCl_3_.7H_2_O bath, respectively. The concentration of CeCl_3_.7H_2_O (60 000 ppm) was optimized in a previous study [21,22]. Figure 1*a,b* shows that a non-uniform white layer developed during the 10 and 30 min of immersion in the CeCl_3_.7H_2_O bath. A covered and uniform white protective film was deposited on the specimens during the 60 and 90 min of immersion, as shown in figure 1*c,d*. However, some traces of cracks can be observed in figure 1*d*.

**Figure 2.**
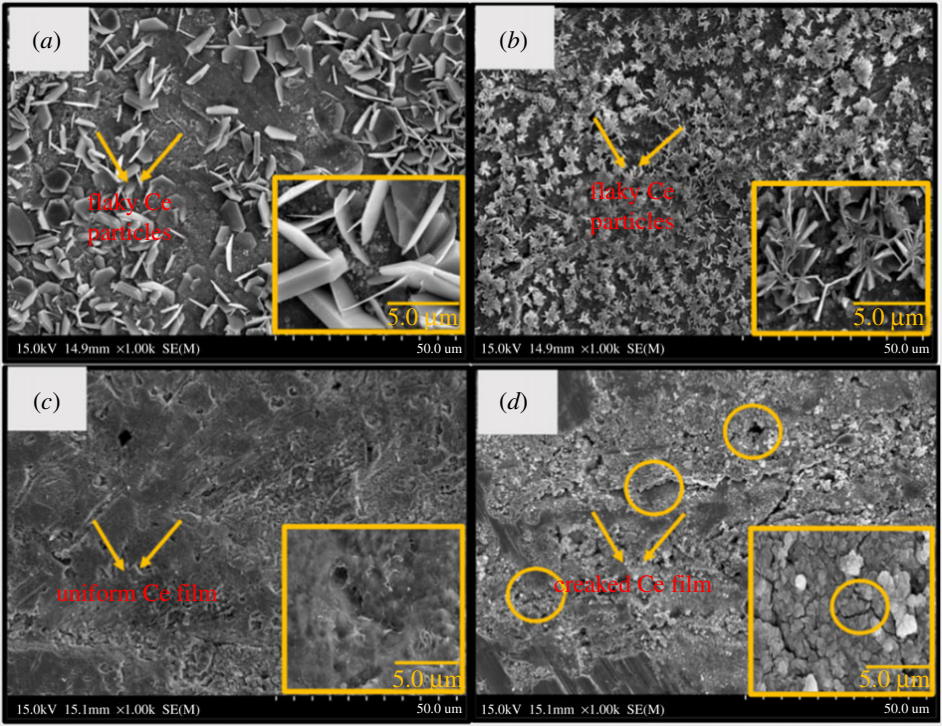
SEM micrographs of (*a*) specimen SA after 10 min, (*b*) specimen SB after 30 min, (*c*) specimen SC after 60 min and (*d*) specimen SD after 90 min.

**Figure 3.**
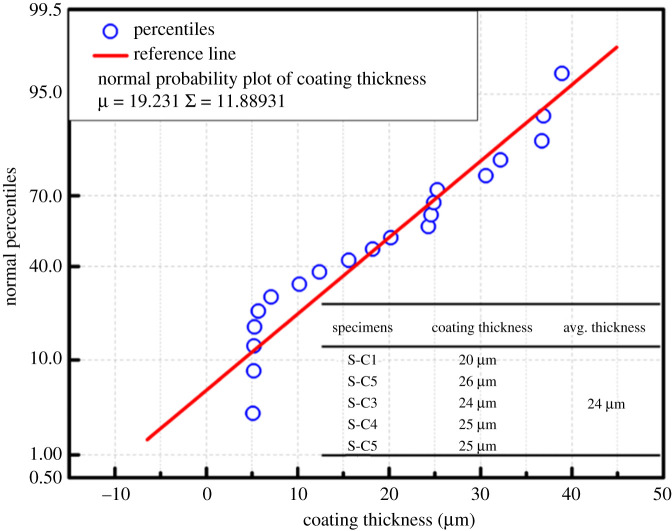
Normal plot for a coating thickness of CeCl_3_.7H_2_O-coated specimens.

The morphology of the protective film was further confirmed by SEM micrographs. Figure 2 depicts the detailed surface morphologies of specimens SA_,_ SB, SC and SD. As shown in figure 2*a,b*, the protective films formed after the 10 and 30 min of immersion in the CeCl_3_.7H_2_O bath do not cover the entire surface continuously. The protective layer, which was thin and non-uniform, probably did not have sufficient time to cover the surface of the specimens continuously and uniformly. Figure 2*c,d* depicts the SEM micrographs of specimens SC and SD. The micrographs of the sealing coating obtained after higher immersion times (60 and 90 min) suggest thick and dense protective films. The specimen SC (figure 2*c*) shows a thicker and uniform protective film on the substrate. Specimen SD (figure 2*d*) shows a uniform and dense layer; however, some cracks can be observed in the protective film. This may be because of the stress developed due to the formation of a thicker sealing coating. This internal stress resulted in an increased propagation of cracks on the surface of the specimens. Larger cracks lead to a higher corrosion rate. The SEM micrographs indicate that specimen SC (figure 2*c*), which was immersed for 60 min in the sealing coating bath, showed the best results. The thickness of the deposited sealing coatings was measured for all the specimens to make the process more scalable. Additionally, if the deposition is carried out further for the same application by following chemical deposition techniques, the optimized thickness measured here will be treated as a baseline. The normal plot (figure 3) shows that the measured sealing coating thicknesses of all the specimens were approximately normally distributed. Specimen SC had an average coating thickness of 24 µm. Moreover, EDS was performed to understand the elemental composition and weight (%) of the elements present on the sealing coating surface.

**Figure 4.**
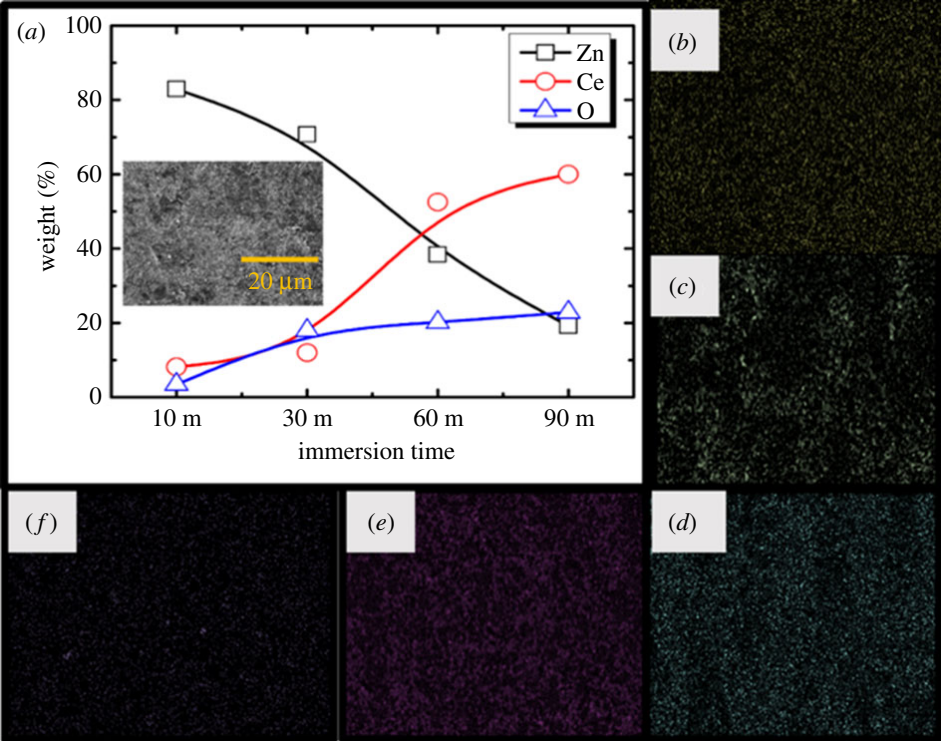
(*a*) SEM image of the coated specimen with corresponding (*b*) EDS weight (%) and EDS maps, (*c*) O, (*d*) Cl, (*e*) Ce and (*f*) Zn.

Figure 4 shows the EDS analysis results of the coated specimen. Based on the EDS mapping results, the sealing coating is composed of Fe, Zn, Ce, O and Cl. The Ce and O content increases in the sealing coating film with an increase in the immersion time. The significant reduction in the concentration of Zn with an increase in immersion time indicated in figure 4*a* on the coated film confirms the presence of Ce-enriched thick and condensed protective film in the coated specimens. The weight (%) of Ce increases by approximately 85.4% after 60 and 90 min of immersion as compared with 10 and 30 min of immersion in the sealing coating bath. Consequently, weight (%) of Zn decreases by approximately 65.2% after 60 and 90 min of immersion as compared with first day and fourth day of immersion in the sealing coating bath.

**Figure 5.**
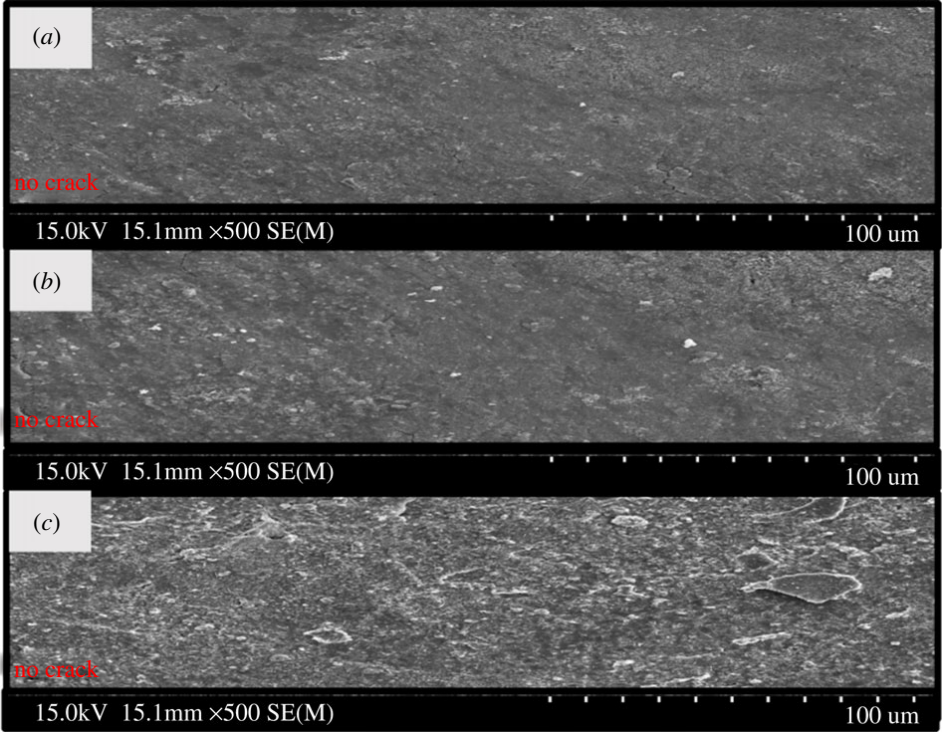
SEM micrograph of coated specimens (SC) studied under various temperatures (*a*) 0°C, (*b*) 25°C and (*c*) 50°C.

Furthermore, the effect of higher temperature on the microstructure of Ce-coated specimens was studied at 0°C, 25°C and 50°C. The above characterizations confirmed the formation of a thick, dense and fully covered protective film for the optimized SC specimen. The temperature study aims to check the suitability of optimized CeCl_3_.7H_2_O sealing coatings on outdoor high-voltage insulators, which face high-temperature variance. The microstructures of the specimens were observed through SEM micrographs, as depicted in figure 5. The SEM analysis indicated that the microstructure of the optimized specimens was not significantly degraded by the temperature change from 0°C to 50°C. No trace of change in the sealing coating colour or cracks can be observed in figure 5. This may be owing to the higher crack resistance and thermal resistance of the sealing coating material. Proper pre-treatment consisting of degreasing and desmutting processes may also have augmented the above characteristics [22–24].

**Figure 6.**
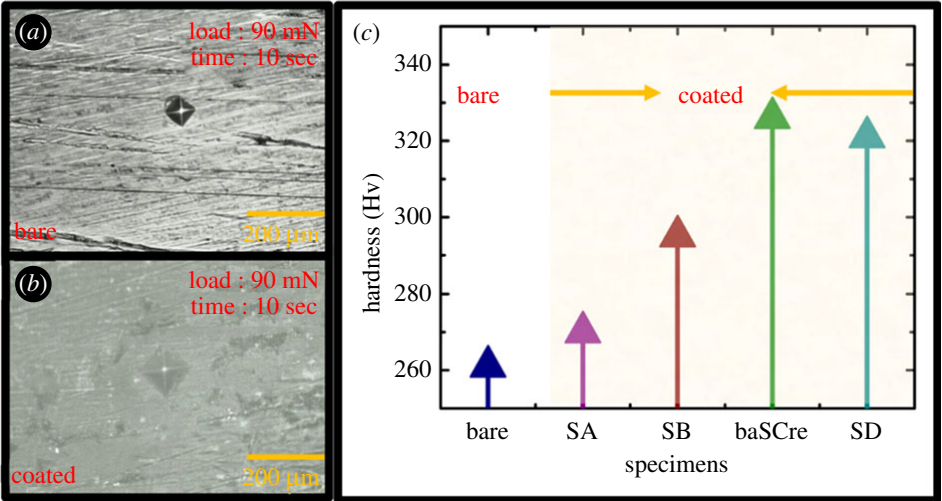
Microhardness analysis of (*a*) bare galvanized specimen, (*b*) CeCl_3_.7H_2_O-coated specimen SC and (*c*) all specimens: bare, SA (10 min), SB (30 min), SC (60 min) and SD (90 min).

A microhardness test was conducted to evaluate the strength, ductility and wear resistance of the coated specimens in harsh outdoor environments. The microhardness values of the bare and coated specimens are shown in figure 6. The hardness of the coated specimens was 16.09% higher than that of the bare specimens, as shown in figure 6*c*. The specimen SC immersed for 60 min in the sealing coating bath exhibited a maximum hardness of 326 HV. However, specimen SD exhibited a 1.5% decrease in hardness with respect to specimen SC. This shows that an appropriate immersion time in the sealing coating bath can enhance the hardness of the specimens. Therefore, the mechanical strength of the coated specimens increased. This was probably because of the refined grain size, crack-free, compact and uniform formation of the protective film [25]. However, an excessive immersion period could have degraded the homogeneity and crack resistance of the specimens, leading to a decrease in the hardness of specimen SD.

**Figure 7.**
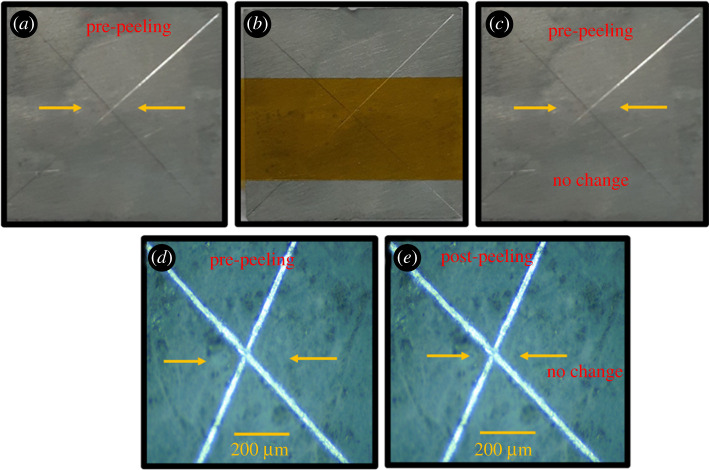
Results of the X-cut tape test of specimen SC (60 min) (*a*) X-cut on specimen SC, (*b*) taped X-cut of specimen SC, (*c*) tape peeled from X-cut of specimen SC, (*d*) optical micrographs of pre-peeled specimen SC and (*e*) optical micrographs of post-peeled specimen SC.

Additionally, the X-cut tape test and two-dimensional finite-element method were performed to evaluate the adhesive bonding and interface strength of the coating–substrate system to prevent premature interface failure. The X-cut tape test of the specimen SC is shown in figure 7. The adhesive strength of the sealing coating and substrate (SC) was 5A level according to ASTM-D3359 standards, which indicates an excellent combination of metal matrix and protective film. Adhesion is rated according to the ASTM standards and the scale varies from 0A (Removal beyond the area of incision) to 5A (No peeling or removal). Table 2 shows the adhesion rating scale for metal substrates. Figure 7*c,e* confirms that specimen SC does not show peeling of the sealing coating film and thereby passes the adhesive test with the 5A scale. Wang *et al*. adopted the D3359 standard to evaluate the adherence strength of the conversion sealing coating and metal matrix. Meanwhile, Wang *et al*. reported that the addition of Ce salt refines the grain size, enhances the crack resistance, reduces the pin holes and develops a compact and uniform protective film. This enhances the bonding strength between the substrate and the sealing coating film [26–30]. Furthermore, simulations were performed to evaluate the interface strength of the coating–substrate system.

**Table 2.** Adhesion rating scale.

scale	criteria
5A	no peeling or removal
4A	trace peeling or removal
3A	rugged removal along incision (1.6 mm)
2A	rugged removal along incision (3.2 mm)
1A	removal from most of the area X under tape
0A	removal beyond the area of the X

**Figure 8.**
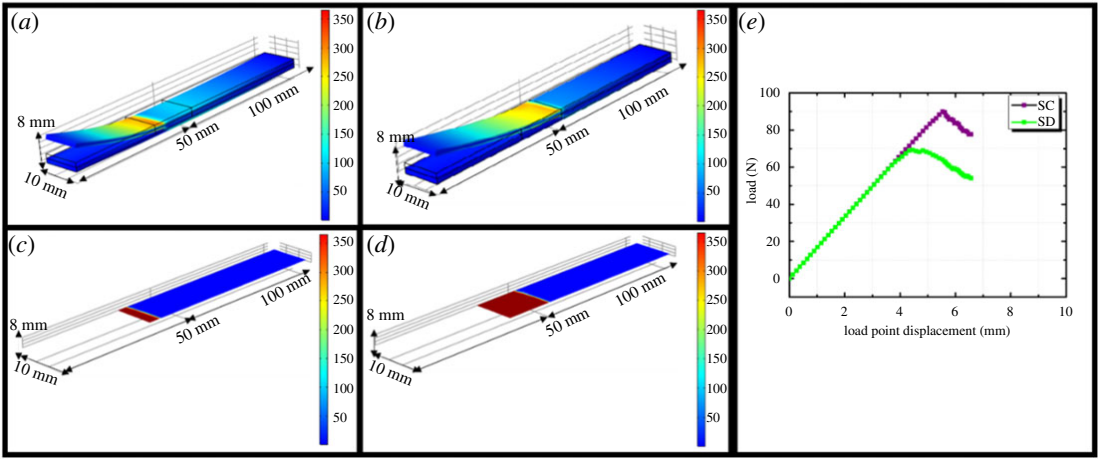
Von Mises stress distributions: (*a*) specimen SC, (*b*) specimen SD, (*c*) interface health of specimen SC, (*d*) interface health of specimen SD and (*e*) load–displacement curve for MMB modelling at 50% mixed-mode loading.

The von Mises stress distribution and load displacement of the specimens is shown in figure 8. A two-dimensional finite-element model implementing the cohesive zone elements was developed to anticipate the interfacial strength and failure through debonding in a coating–substrate system. The approach adopted cohesive zone modelling with the bilinear traction separation law. The material properties required for the constitutive model of the coating–substrate system are summarized in table 3. The CZM is defined using the bilinear traction separation law. The traction increases linearly with stiffness before the opening crack reaches the damage initiation displacement. When the crack opens beyond the damage initiation replacement, the coating material softens, and the stiffness increases as a function of damage *d*. The coating–substrate system fails if the stiffness decreases to 0. Here, the mixed-mode bending (MMB) model by Benzeggah and Kenane was used to simulate the interface strength and failure of specimens.

**Table 3.** Properties of the coating and substrate used in simulation.

property	adherend	adhesive
normal tensile strength (MPa)	510	100
shear strength (MPa)	11.81	1.33
Young's modulus (GPa)	122.7	28
Poisson's ratio	0.3	0.24
shear modulus (GPa)	3.70	10
density (g cm^−3^)	7	6.6
microhardness_1_ (HV): SC	326	
microhardness_2_ (HV): SD	320	
penalty stiffness (N mm^−3^)	1 000 000	

The MMB modelling considered an initial crack of length C_1_ (5 mm) along the interface of the coating–substrate system. A beam at both ends supported the specimen. A MMB load was generated because of the forces applied to the edges closer to the cracked end and at the centre of the specimen. Two levers were used to transmit the force to the specimens. The first lever, which applied the pushing down force Fm, was placed at the centre of the specimen. Another lever was attached to the cracked end of the specimen, which transmitted the pulling force Fe on the cracked side of the specimen. The model was studied for a mixed-mode ratio of 50%. The initial crack propagated along the interface in both the specimens owing to the application of force in both specimens SC and SD, as shown in figure 8*a,b*. In specimen SC (figure 8*a*), the interface crack extended to approximately 45 mm. The interface health of specimen SC (figure 8*c*) depicts the maximum stress distribution near the crack end. However, in the case of specimen SD (figure 8*b*), the initial crack propagated beyond 50 mm along the interface.

The interface health of specimen SD (figure 8*d*) shows that the maximum stress is concentrated from 45 to 53 mm along the interface of specimen SD. The reason for the poor interface health of SD may be the presence of cracks in the sealing coating layer, as confirmed by the SEM observations. These cracks could have led to the interface failure of the coating–substrate system. The load–displacement curve can be plotted using this simulation study, as depicted in figure 8*e*. If the load applied to the specimens increases the maximum load capability of the specimens with an initial crack, interface failure or delamination occurs. After increasing the load to the peak value, the load decreased until the displacement reached approximately 7 mm for both specimens. This indicated when the crack reached the middle of the specimen, after which the load began to increase again with a much lower stiffness compared with the pre-delamination state.

**Figure 9.**
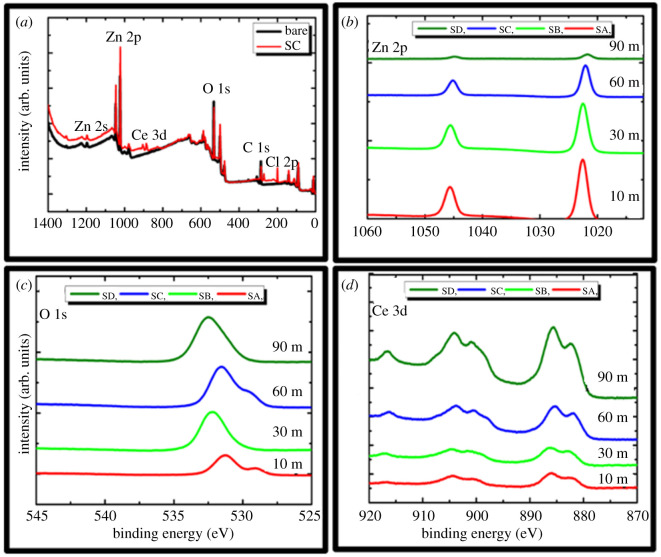
Results of XPS analysis (*a*) comparison of bare and coated specimens, (*b*) narrow spectra analysis of Zn 2p, (*c*) narrow spectra analysis of O 1s and (*d*) narrow spectra analysis of Ce 3d.

XPS analysis was used to analyse the surface binding energy of the protective film. The compositions of the protective films of specimens SA, SB, SC and SD were characterized by XPS after immersion in industrial media (Sol-I4). Figure 9shows the survey results and the significant narrow spectra of the evaluated specimens post immersion to industrial media (Sol-I4). The main elements found were Zn, O, Ce, C and Cl, as shown in figure 9*a*. The presence of C is due to the surface contamination. The XPS spectra of Zn 2p in figure 9*b* depict the presence of a peak at approximately 1021.7 eV, which represents the oxidized state of the ZnO-type compound. This spectrum remained constant at this binding energy throughout all the specimens, as observed in figure 9*b*. The high-resolution XPS spectrum for O (figure 9*c*) illustrates a single peak, which may be attributed to both Zn–O and Ce–O compounds. This peak is situated at a binding energy of 532.16 eV and is associated with the presence of ZnO, as it has a binding energy of 531.5 ± 1 eV. Simultaneously, the peak may be associated with the formation of hydrated Ce oxide/hydroxide. The peaks associated with the hydroxylated groups possess binding energies of 532.2 eV. Moreover, binding energies associated with Ce_2_O_3_ also exist in the same energy range, i.e. 530.3 eV. The XPS spectra of Ce 3d (figure 9*d*) appeared in three different zones. The first zone corresponds to the peaks that appear in the binding energy range of 880–890 eV (Ce 3d_5/2_). Similarly, the second zone corresponds to the binding energies in the range 890–910 eV; the Ce 3d_3/2_ and Ce 3d_5/2_ spectra overlap in this zone. The third zone exists because of the peak associated with Ce 3d_3/2_ at an energy of 917 eV. A comparison of the theoretical values of the Ce spectra revealed that Ce(OH)_3_, CeO_2_ and Ce_2_O_3_ were present in the protective films. Table 4 summarizes the atomic weights (%) of the important elements confirmed by the XPS analysis.

**Table 4.** Atomic weight (%) of vital elements detected in the surface of the sealing coating.

sample (min)	Zn 2p (%)	O 1s (%)	Ce 3d (%)
10	26.95	30.2	0.8
30	23.91	40.21	1.91
60	11.64	42.4	4.48
90	2.05	46.2	8.25

**Figure 10.**
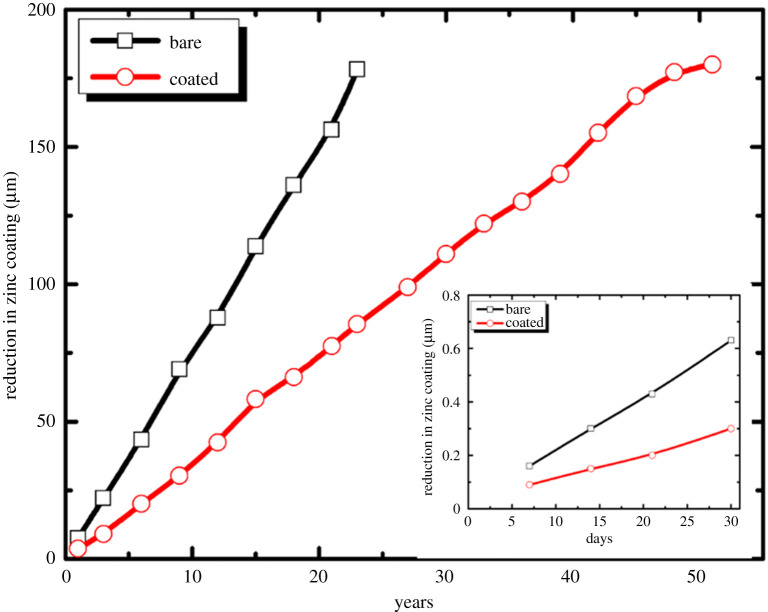
Life expectation of bare and coated insulator pin (SC) installed in low-pH industrial sites.

It was observed that with an increase in the immersion time (10–90 min), the atomic weight (%) of Zn 2p degraded 13 times. Simultaneously, the O 1s and Ce 3d peaks rose by 1.5 and 10 times, respectively. The Zn 2p and Ce 3d peaks were attributed to ZnO and Ce compounds. With the formation of the Ce-enriched protective film, the atomic weight (%) of Ce compounds increased and ZnO decreased on the surface. The O 1s peak is attributed to ZnO, Ce(OH)_3_, CeO_2_ and Ce_2_O_3_. Thus, the atomic weight (%) of O increased with the increase in the immersion time. The XPS results (figure 9*a*) confirm 1.3 times higher intensities of surface binding energy for coated specimens in contrast with the bare specimens. This implies that more energy is required to knock out the atoms from the surface in the coated specimens than that required in the bare ones. This enhanced the reliability of the coated specimens. After coating synthesis, optimization and evaluation of the optimized coating by implementing the various characterization techniques, it is necessary to determine the life expectancy of the sealing coating applied pin specimens. Figure 10 and electronic supplementary material, figures S1, S2, S3 and table S1, depict the life expectancy of the galvanized coating on an insulator pin associated with a high-voltage device installed in highly corrosive industrial sites, with a corrosion rate of 5.58–12 µm yr^−1^. The Zn reduction in all the corrosive media (Sol I1–I4) was observed as approximately 0.62 and 0.3 µm month^−1^ for the bare and coated specimens (SC), respectively. A decrease of approximately 51.6% in the Zn film etching can be observed in the coated specimens (SC) compared with the bare specimens. Considering the observations, the life expectancy was calculated for the bare and coated specimens after exposure to the industrial media. The life expectancy of galvanized coating for bare and coated specimens (SC) was calculated as approximately 20 years and 50 years, respectively. Ce-based sealing coatings are suitable for use as anti-corrosion coatings for high-voltage insulator pins with an expected lifespan of 30–50 years.

Ce compounds are widely investigated for future thermal barrier coatings and hydrophobic coatings owing to their high melting point (1060 K), low thermal conductivity (2 W mK^−1^) and easily tailored structures [28–32]. Interest in Ce-based anti-corrosion approach has increased in recent decades owing to their various positive characteristics. However, the synthesis approach adapted in the article is noble and such green coatings have not been implemented in insulator hardware/power utilities yet. The article describes implementation of sealing coating on insulator pin. However, it can be implemented on other metal parts prone to corrosion in power industries in future.

## 4. Conclusion

The Ce-based sealing coatings produced on galvanized pin specimens were examined in this study to meet the dependability standards of the coating industry. The OM and SEM findings indicated that specimens submerged in the sealing coating bath for 60 min, displayed thick, dense and homogeneous protective coatings. The EDS data show that the sealing layer contained Fe, Zn, Ce, O and Cl. Furthermore, the SEM micrographs show that temperature fluctuations had no effect on the microstructure of the coated specimens. This might be due to the sealing coating material's increased fracture and temperature resistance. The Vickers hardness test found that the coated specimens had a 16.09% increase in microhardness when compared with the untreated specimens. According to the ASTM-D3359 standards, the adhesive strength of the sealing coating and substrate (SC) was measured as 5A in the X-cut tape test, indicating an outstanding combination of metal matrix and protective film. The presence of Ce(OH)_3_, CeO_2_ and Ce_2_O_3_ in the protective coatings was established by XPS. The galvanized coating's life expectancies for untreated and coated specimens were determined to be roughly 20 and 50 years, respectively. All the above discussions prove CeCl_3_.7H_2_O to be an eco-friendly, economical and reliable coating material. Thus, it can be concluded that Ce-based optimized sealing coatings are suitable for application on galvanized insulator pins associated with electrical insulators with a minimum lifespan of 30 years. Super-hydrophobicity, self-cleaning and anti-microbial qualities can be created by adjusting the sealing coating development process to broaden the application in future. We hope that this research will pave the way for the development of environmentally acceptable anti-corrosion coatings for power sectors.

## Data Availability

Data is shared through supplementary material and stored in figshare [33].
